# Deletion of *Gtf2i* via Systemic Administration of AAV-PHP.eB Virus Increases Social Behavior in a Mouse Model of a Neurodevelopmental Disorder

**DOI:** 10.3390/biomedicines11082273

**Published:** 2023-08-15

**Authors:** Omer Ophir, Gilad Levy, Ela Bar, Omri Kimchi Feldhorn, May Rokach, Galit Elad Sfadia, Boaz Barak

**Affiliations:** 1 The Sagol School of Neuroscience, Tel Aviv University, Tel Aviv 6997801, Israel; 2 The School of Psychological Sciences, Faculty of Social Sciences, Tel Aviv University, Tel Aviv 6997801, Israel; 3 The School of Neurobiology, Biochemistry & Biophysics, Faculty of Life Sciences, Tel Aviv University, Tel Aviv 6997801, Israel

**Keywords:** Williams syndrome (WS), *Gtf2i*, adeno-associated virus (AAV), PHP.eB serotype, post-natal deletion, excitatory neurons, social behavior

## Abstract

Williams syndrome (WS) is a neurodevelopmental disorder characterized by distinctive cognitive and personality profiles which also impacts various physiological systems. The syndrome arises from the deletion of about 25 genes located on chromosome 7q11.23, including *Gtf2i*. Prior research indicated a strong association between pre-natal *Gtf2i* deletion, and the hyper-social phenotypes observed in WS, as well as myelination deficits. As most studies addressed pre-natal *Gtf2i* deletion in mouse models, post-natal neuronal roles of *Gtf2i* were unknown. To investigate the impact of post-natal deletion of neuronal *Gtf2i* on hyper-sociability, we intravenously injected an AAV-PHP.eB virus expressing Cre-recombinase under the control of αCaMKII, a promoter in a mouse model with floxed *Gtf2i*. This targeted deletion was performed in young mice, allowing for precise and efficient brain-wide infection leading to the exclusive removal of *Gtf2i* from excitatory neurons. As a result of such gene deletion, the mice displayed hyper-sociability, increased anxiety, impaired cognition, and hyper-mobility, relative to controls. These findings highlight the potential of systemic viral manipulation as a gene-editing technique to modulate behavior-regulating genes during the post-natal stage, thus presenting novel therapeutic approaches for addressing neurodevelopmental dysfunction.

## 1. Introduction

Williams syndrome (WS) is a neurodevelopmental disorder characterized by distinct cognitive and personality profiles which affects various systems [1]. Individuals with WS exhibit intellectual disability, heightened sociability, increased empathy, and elevated anxiety related to specific phobias. The syndrome is caused by the haploinsufficiency of approximately 25 genes from the WS critical region (WSCR) located on chromosome 7q11.23 [2,3]. Previous studies demonstrated how perturbation of *Gtf2i*, a gene within the WSCR, led to the social and cognitive impairments [4,5,6,7,8] and myelin alterations [9] observed in WS. Research using animal models offered insight into the functional consequences of *Gtf2i* loss. In mice, the loss of *Gtf2i* function leads to multiple phenotypic manifestations, including embryonic lethality, brain hemorrhage, and defects in vasculogenesis, craniofacial development, and neural tube formation. Heterozygotes of *Gtf2i* display microcephaly, retarded growth, and skeletal and craniofacial defects, indicative of the critical role of Tfii-i, the protein product of *Gtf2i,* dosage’s during embryonic development. Moreover, the down-regulation of *Gtf2i*-associated genes in mice is associated with abnormalities in core biological processes and with skeletal and craniofacial pathogenesis, traits also observed in human WS patients [10]. At the same time, like the majority of mammalian genes, *Gtf2i* presents changing patterns of expression during development, depending on its role at any given time [11]. Moreover, it has been reported that *Gtf2i*-mediated effects are dosage-related. Specifically, while haploinsufficiency of *Gtf2i* leads to WS phenotypes [1,9,12], duplication of *Gtf2i* leads to autistic phenotypes, such as those noted in 7q11.23 duplication syndrome (Dup7) [13,14,15,16,17,18]. Still, the effects of post-natal *Gtf2i* deletion remain largely unexplored, which is the purpose of our current study.

Recent advancements in gene therapy have raised the promise of improved quality of life of patients afflicted with a variety of neurological conditions [19]. The exceptional life cycle of adeno-associated virus (AAV) and its ability to infect both non-dividing and dividing cells while maintaining persistent expression have made it a highly desirable vector in such efforts [20]. Furthermore, the wild-type virus lacks any noticeable pathogenicity [21]. AAV-based post-natal *Gtf2i* gene therapy in a mouse model of WS in which there is complete pre-natal deletion of the WSCR resulted in the restoration of mouse (*m*)*Gtf2i* levels, alongside improved motor coordination and sociability, as well as decreased anxiety, as compared to controls [22].

To explore the consequences of early post-natal deletion of *Gtf2i* from excitatory neurons, we investigated post-natal roles of Tfii-i, the protein product of *Gtf2i*. For this, we recruited a mouse model with *Gtf2i loxP* sites [23,24] and induced post-natal deletion through intravenous (IV) injection of an AAV expressing Cre-recombinase under αCaMKII promoter regulation [25]. We specifically manipulated post-natal *Gtf2i* expression in excitatory neurons. This allowed for comparison with previous efforts addressing pre-natal roles for the gene in excitatory neurons, specifically mediating myelination development [26,27] and affecting behavior [9]. Such a comparison of *Gtf2i* pre-natal and post-natal neuronal functions would enable us to better understand the critical developmental window in WS in which genetic manipulation would be effective on both brain development and behavior. Moreover, the rationale for exploring the impact of post-natal *Gtf2i* deletion lies in the potential for developing therapeutic interventions for neurodevelopmental disorders and other pathological conditions beyond current behavioral therapies and pharmaceutical agents.

## 2. Methods

### 2.1. Mice

**Breeding**: Mice that carried homozygous *loxP* sites flanking the *Gtf2i* gene were bred with each other. The offspring were previously shown to exhibit normal behavior and development and possess the background of C57Bl/6 mice. Male mice were used in order to compare the results of this study with our previous study related to prenatal deletion of *Gtf2i* from excitatory neurons, which was also conducted using male mice [9].

**Housing**: Mice of the same sex, regardless of genotype, were housed together in cages containing 2–4 mice. The mice were kept in a controlled environment at a temperature of 20–24 °C and a 12 h light-dark cycle (lights on at 07:00, lights off at 19:00) and had access to food and water ad libitum. All experimental procedures followed the guidelines of the Institutional Animal Care and Use Committee of Tel Aviv University, Tel Aviv, Israel. Every effort was made to minimize animal suffering and reduce the number of animals used.

### 2.2. Injection

P1–2 male mice were first lightly anesthetized on ice before injection into the superficial temporal facial vein [25] using a Nanofil-100 syringe and a 34G beveled needle with 30 μL of 10^12^ vg/mL of the AAV virus.

### 2.3. DNA Extraction

Tissue samples were suspended in 100 µL alkaline lysis buffer (25 mM NaOH (BioLab, Jerusalem, Israel) and 0.2 mM EDTA (Sigma-Aldrich, St. Louis, MO, USA) diluted in DDW, pH 12) for 30 min at 95 °C while shaking at 600 rpm. To terminate the lysis reaction, 100 µL of neutralization buffer (40 mM Tris-HCl (Sigma-Aldrich) in DDW, pH 5) was added to the sample, and the mix was cooled at 4 °C for at least 5 min.

### 2.4. Polymerase Chain Reaction (PCR)

To determine the sex and genotype (GT) of each mouse, PCR was conducted. For GT, a mixture of 8 µL DDW, 12.5 µL DreamTaq Green PCR Master Mix (2×) (Thermo Fisher Scientific, Waltham, MA, USA), and 0.5 µL of each of the 3 Nex-Cre primers (1.5 µL in total; primers were ordered from Hy-Laboratories (Rehovot, Israel) and diluted to 10 mM according to the manufacturer’s instructions). The mixture was added to the PCR tube, followed by 3 µL DNA extraction from each mouse. A C1000 Touch thermal cycler (Bio-Rad, Hercules, CA, USA) was used under the following conditions: 95 °C for 4 min, 30 amplification cycles containing three temperature steps (denaturing at 94 °C for 30 s, annealing at 55 °C for 30 s, and elongation at 72 °C for 1 min), followed by 7 min at 72 °C and holding at 4 °C until the end. For sex determination, a mixture of 6.5 µL DDW, 12.5 µL DreamTaq Green PCR Master Mix (2×), and 0.5 µL of each of the 2 XY primers (1 µL in total; primers were ordered from Hy-Laboratories) was diluted to 10 mM according to the manufacturer’s instructions. The mixture was added to the PCR tube, followed by 5 µL DNA extraction from each mouse. A C1000 Touch thermal cycler was used under the following conditions: 94 °C for 2 min, 30 amplification cycles containing three temperature steps (denaturing at 94 °C for 20 s, annealing at 60 °C for 20 s, and elongation at 72 °C for 30 s), followed by 5 min at 72 °C and holding at 4 °C until the end. For primer sequences, see Table 1.

### 2.5. Gel Electrophoresis

To determine mouse sex and GT, 8.5 µL from each PCR product was added to a well of a 2% agarose gel (1× TAE (Bio-Lab, Poole, UK), 2% agarose (Hy Laboratories), 3% Serva DNA Stain Clear G dye (Serva Electrophoresis, Heidelberg, Germany), alongside a 100 bp ladder (DM2100 ExcelBand, Smobio Technology, Hsinchu, Taiwan). The GT of these mice was determined by a single band (~770 bp). Males presented two bands (269 and 353 bp), while females presented a single band (269 bp). The sex determination primers and protocol were adapted from Tunster (2017) [28].

### 2.6. Behavioral Tests

All behavioral studies were conducted and analyzed in a double-blind fashion, with the experimenter unaware of the genotypes. Male mice were exclusively selected for the experiments and were acclimated in the designated test room for an hour before each behavioral assessment. Each cohort of test mice underwent a maximum of four behavioral tests, with a minimum inter-test interval of three days.

**Social interaction and novelty test:** To conduct the test, C57-black mice of similar ages and body weights were obtained from the Jackson Laboratory. A habituation procedure was carried out to familiarize the mice with unfamiliar individuals for the social preference test. This involved placing the mice inside an inverted wire cup for 30 min, twice a day, over a period of three consecutive days prior to the test.

The test apparatus used had dimensions of 65 cm (length) × 44 cm (width) × 30 cm (height) and was divided into three sections, each measures 21 cm × 44 cm. The sections were interconnected by a lever-operated door positioned 5 cm above the floor. The objects of interest, including a stranger mouse and a familiar mouse, were placed inside separate inverted wire cups (10 cm high, with a bottom diameter of 10 cm and bars spaced 0.8 cm apart). Additionally, a weighted cup was positioned on top of the wire cup set up to prevent the test mice from climbing over it. Following each trial, the wire cup and equipment were cleaned using a combination of ethanol and water.

The test itself consisted of three distinct phases, each lasting 15 min: habituation, sociability, and social novelty. During the habituation phase, the test mouse was placed in the center section and allowed to explore the entire apparatus with the wire cups empty. Subsequently, in the sociability test, the test mouse was placed in the center section with the doors closed, while a stranger mouse and an object were positioned in the wire cups of the left and right sections. Afterward, the doors were lifted to enable the test mouse to freely explore the environment. Finally, in the social novelty test, the test mouse was once again placed in the center section with the doors closed, but this time a novel mouse was introduced instead of an object. Once again, the doors were raised to allow the test mouse to explore the setup. The positions of the object and the stranger mouse were alternated between trials to prevent any potential bias based on location preference.

To ensure controlled stimulation, each of the stimulation mice was used a maximum of two times per day. Following the completion of the test, the experimenter, who remained unaware of the treatment type, analyzed the time spent with the object as compared to the stranger mouse, and the time spent with the familiar mouse compared to the novel mouse. Analysis was performed using EthoVision XT 14.0.1326 software (Noldus Information Technology, Wageningen, The Netherlands).

**Open-field exploration test:** Each mouse was placed individually in the center of a Plexiglas box measuring 40 cm in length, 40 cm in width, and 30 cm in height for a duration of 30 min. The purpose of this test was to assess motor activity and exploration behavior. Video recordings were made to capture mouse movements, specifically focusing on the time spent in the peripheral areas of the box. Importantly, the experimenter conducting the analysis remained unaware of the treatment administered to the mice.

**Elevated zero maze:** The elevated zero maze utilized in this experiment consisted of two distinct sections, namely, open and closed arms. The entire maze was 60 cm high. Mice were initially placed in the closed arms of the maze and allowed to explore for five minutes. The objective of this test was to evaluate the time spent by the mice in the open arms, which serves as an indicator of anxiety-like behavior. The movement of the mice between the sections was recorded using video cameras. Like previous tests, the experimenter assessing the results remained blind to each mouse’s specific treatment.

**Rotarod:** Mice were placed on an accelerating rotarod apparatus to assess their motor coordination, measuring the latency to fall. Each mouse underwent three separate trials, with a time interval of 30 min between each test.

### 2.7. Brain Tissue Extraction

Following the completion of the behavioral experiments, male mice were deeply anesthetized using isoflurane. The level of consciousness was assessed by observing the response to a foot pinch. Tissue samples were collected from one ear for genetic verification purposes. Subsequently, the mice underwent perfusion with 15 mL of an ice-cold PBS solution. This was followed by brain dissection, wherein the brain was carefully separated into two hemispheres.

One hemisphere was placed in a solution of 4% paraformaldehyde (PFA) diluted in PBS and kept for 48 h to facilitate proper fixation. The other hemisphere was placed in a Petri dish containing PBS for dissection purposes. Using sterilized surgical tools and a stereomicroscope (Olympus, Tokyo, Japan), the cortex was dissected from the remaining brain tissue. To preserve the integrity of the cortical samples, they were placed in 200 µL of RNA-later solution (Invitrogen, Carlsbad, CA, USA) and kept at 4 °C for 24 h. Following this preservation period, the RNA-later solution was removed, and the samples were frozen at −80 °C. Alternatively, the samples were directly placed into liquid nitrogen and subsequently frozen at −80 °C.

To ensure the sterility and integrity of the experimental process, all tools and equipment used were sterilized with ethanol and treated with RNAse inhibitors (RNase-ExitusPlus, Biological Industries, Kibbutz Beit-Haemek, Israel).

### 2.8. RNA Isolation and qPCR

**RNA extraction:** Cortices were placed in safe-lock tubes along with stainless steel beads and 500 µL of cold TRIzol reagent (Thermo Fisher Scientific). Homogenization was achieved using the TissueLyser 2 (Qiagen, Hilden, Germany) for 60 s at a frequency of 24,000 Hz. Once the tissue was fully homogenized, an additional 500 µL of TRIzol was added, and the samples were incubated at room temperature (RT) for five minutes. Subsequently, 200 µL of chloroform (Bio-Lab) was added to each tube, followed by manual shaking for 15 s and an additional three-minute incubation at RT.

The samples were then centrifuged for 20 min at 4 °C and 800× *g* (13,800 rpm) using an Eppendorf 5430R Centrifuge. The centrifugation resulted in the separation of the homogenate into protein, DNA, and RNA layers, with the RNA layer on top. The clear RNA layer was carefully transferred to new tubes and diluted in a 1:1 proportion with isopropanol (Bio-Lab). The tubes were manually shaken, incubated for five minutes at RT, and then centrifuged for 15 min at 4 °C and 800× *g* (13,800 rpm). The centrifugation step precipitated the RNA, and the isopropanol was subsequently removed. The remaining RNA pellet was washed twice, each time with 1 mL of 80% ethanol (Sigma-Aldrich) diluted in DEPC-treated water (Biological Industries), followed by another round of centrifugation for 15 min at 4 °C and 13,800 rpm.

After the washes, all ethanol was carefully removed, and the tubes were opened to allow the remaining ethanol to evaporate for approximately 30 min. To resuspend the RNA, 35 µL of DEPC-treated water was added to each sample, followed by heating for five minutes at 60 °C. Finally, the samples were pipetted to ensure a homogeneous concentration, and the concentration was measured using a Thermo Scientific NanoDrop One device (Thermo Fisher Scientific). The extracted RNA was then frozen at −80 °C.

**Complementary deoxyribonucleic acid (cDNA) preparation:** After RNA extraction, the obtained RNA samples were diluted to a concentration of 100 ng/µL. The reverse transcription process was performed using random primers and the High-Capacity cDNA Reverse Transcription Kit (Thermo Fisher Scientific). The following protocol was employed using the C1000 Touch thermal cycler: an initial step of 10 min at 25 °C, followed by 120 min at 37 °C, 5 min at 85 °C, and a final step at 4 °C. The resulting cDNA was then frozen at −20 °C for further use.

**Real-Time PCR:** mRNA levels were measured by real-time PCR with the Fast SYBR Green PCR Master and the Bio-Rad CFX Connect Real-Time PCR Detection System. The PCR protocol consisted of an initial denaturation step at 95 °C for 20 s, followed by 40 amplification cycles. Each cycle included denaturation at 95 °C for 3 s, annealing, and extension at 60 °C for 30 s. Additionally, a melt curve analysis was performed by subjecting the samples to 60 °C for 5 s, followed by an increase of 0.5 °C every 5 s (using a heated plate reader) until reaching 95 °C.

To determine mRNA levels, the comparative cycle threshold (Ct) method was used [29]. The mRNA expression of the target genes was normalized to the mRNA expression of glyceraldehyde 3-phosphate dehydrogenase (Gapdh). The results were presented as fold change (FC) relative to the control group.

Primers were from Hy Laboratories. Primers were diluted with 10 mM in DEPC-treated water (Table 1).

### 2.9. Immunofluorescent Staining

Brains were extracted as previously described and then sectioned at a thickness of 100 µm using a vibratome (Leica Biosystems, Wetzlar, Germany). For staining, the free-floating method was employed as follows: A section from the motor cortex, approximately located at bregma 1.18 mm according to the mouse atlas, was selected for further analysis. The sections were washed three times in 1 mL PBS for five minutes each. Subsequently, the sections were permeabilized with 1.2% Triton X-100 in PBS for 15 min. Following permeabilization, the sections were washed three times in 1 mL of PBS for five minutes each and then blocked with a solution containing 5% normal goat serum (NGS), 2% bovine serum albumin (BSA), and 0.2% Triton X-100 in PBS for an hour.

After blocking, the sections were placed in a 96-well plate and incubated overnight at 4 °C with primary antibodies diluted in a blocking buffer. The next morning, the sections were washed three times in 1 mL of PBS for 10 min each time. Subsequently, the sections were incubated with secondary antibodies conjugated with Alexa Fluor 488, 555, and 647 (1:1000; catalog nos. A11001, A21424; Invitrogen) diluted in a blocking buffer for an hour. After another round of washing (three times in 1 mL of PBS for 10 min each), the sections were mounted on glass slides using VECTASHIELD Hard-set Antifade Mounting Medium with DAPI (catalog no. H-1500-10, Vector Laboratories, Newark, CA, USA).

Image acquisition was performed using a light microscope (IX-83, Olympus) with the experimenter blinded to the treatment type. For quantification of cellular properties in the motor cortex, images were captured at ×10, ×20, or ×40 magnification, depending on the staining and analysis requirements. Cell numbers were manually quantified using the ImageJ program.

Characterization of viral transfection properties was performed by analyzing cells from the secondary motor cortex (M2), which were imaged from coronal brain sections at a 1.18 mm bregma.

Commercial antibodies used: anti-mCherry (1:1000, catalog no. ab205402, Abcam, Cambridge, UK); anti-NeuN (1:1000, catalog no. MAB-377, Mercury, Fond du Lac, WI, USA); anti-olig2 (1:1000, catalog no. AB9610, Mercury); anti-Iba1 (1:500, catalog no. 234006, SYSY); anti-Gad67 (1:1000, catalog no. MAB5406, Mercury); anti-Gfap (1:1000, catalog no. mab360, Millipore, Burlington, MA, USA); anti-Tfii-i (1:1000, catalog no. CST-4562S, Cell Signaling, Danvers, MA, USA).

### 2.10. Western Blot

Brains were dissected as previously explained and homogenized in solubilization buffer (50 mM HEPES, pH 7.5, 150 mM NaCl, 10% glycerol, 1% Triton X-100, 1 mM EDTA, 1 mM EGTA, 1.5 mM MgCl_2_, and 200 μM Na_3_PO_4_), supplemented with a 1:100 dilution of protease inhibitor cocktail 1 (Merck, Rahway, NJ, USA). Equal amounts of protein from each sample were loaded and resolved by 10% SDS-polyacrylamide gel electrophoresis. The separated proteins were then transferred to a nitrocellulose membrane using transfer buffer composed of 25 mM Tris-HCl (pH 8.3), 190 mM glycine, and 10% (*v*/*v*) methanol. The membranes were subsequently blocked in TBST buffer (0.05 M Tris-HCl, pH 7.5, 0.15 M NaCl, and 0.1% Tween 20) containing 6% skim milk. Following overnight incubation with anti-Tfii-i (1:1000, catalog no. CST-4562S, Cell Signaling) and anti-tubulin (1:5000, catalog no. AB108342, Abcam) antibodies in TBSTX1 buffer for tubulin or blocking buffer for Tfii-i, the membranes were further incubated with horseradish-peroxidase-conjugated secondary antibodies for 40 min. Immuno-reactive bands were detected using an enhanced chemiluminescence reagent.

### 2.11. Statistical Analysis

Data are presented as the mean ± standard error of the mean (SEM), computed using GraphPad Prism 9.4.1 for Windows (GraphPad Software, San Diego, CA, USA). For statistical analysis, *p*-values were calculated utilizing Student’s *t*-test, 1-way repeated-measures ANOVA, 2-way repeated-measures ANOVA, Mann–Whitney test, Wilcoxon matched-pairs signed-rank test and correlation test. A significance level of *p* < 0.05 was considered significant (* *p* < 0.05, ** *p* < 0.01, *** *p* < 0.005, **** *p* < 0.0001).

**Figure 1:**
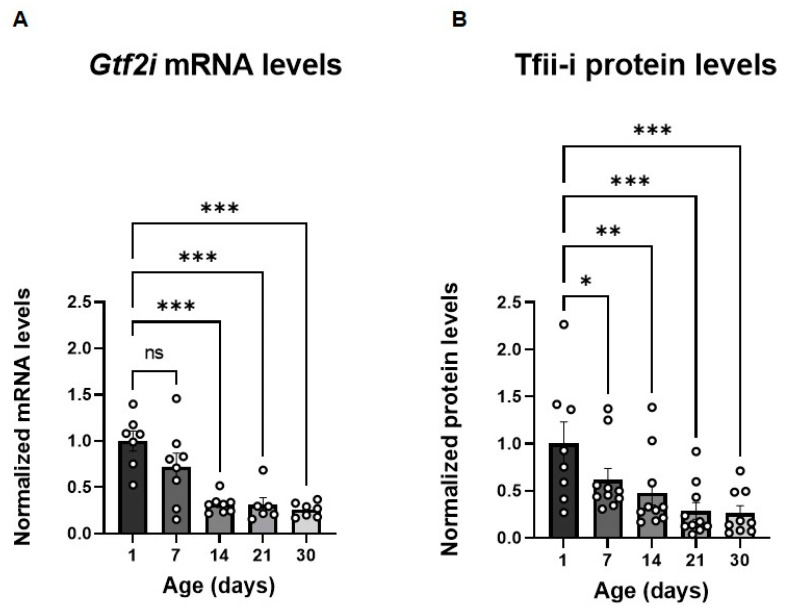
*Gtf2i* mRNA and Tfii-i levels decrease during the first post-natal month. (**A**) *Gtf2i* mRNA levels decrease during development. This becomes significant after 14 days. (**B**) Tfii-i protein levels decrease during development. This becomes significant after 7 days. Statistical significance was determined using two-way ANOVA. *n* = 8 for each age group. (**A**) *p* < 0.0001, F (4, 24) = 11.17. (**B**) *p* < 0.0001, F (4, 33) = 8.286. Tukey’s multiple comparisons test was used for post-hoc analysis. * *p* < 0.05, ** *p* < 0.01, *** *p* < 0.005.

**Figure 2:**
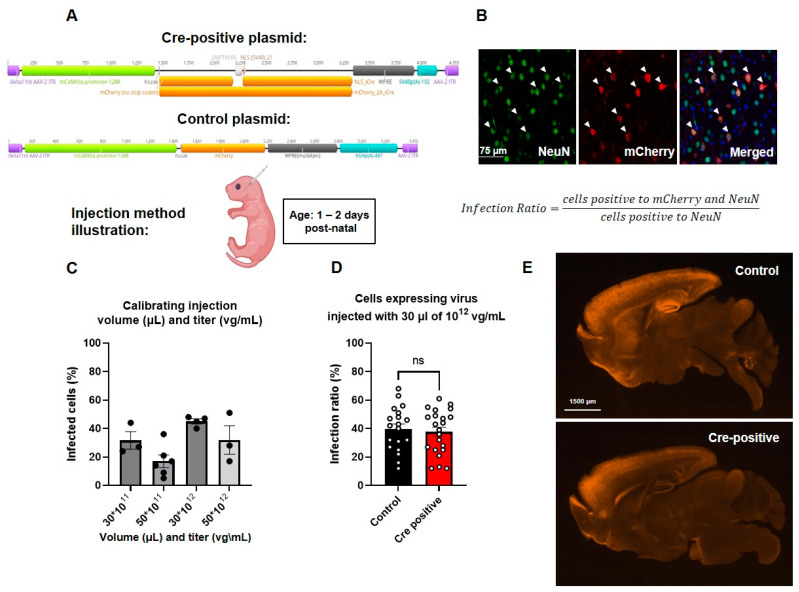
Similar high levels of infection were found in both the Cre-positive and control groups. (**A**) Schematic depiction of the plasmids used. (**B**) Representative images of the viral infection pattern in the mouse brain. Merged image shows NeuN, viral mCherry, and DAPI. (**C**) Infection ratios at different titers (vg/mL) and volumes (µL). (**D**) Infection ratios are similar in mice infected with control and Cre-positive viruses. Unpaired *t*-test, *n* = 19 for control, *n* = 22 for Cre-positive: *p* = 0.674. (**E**) Infection patterns in mid-sagittal brain slices indicating similar infection properties of the two viruses used.

The normality of distributions and equality of variances were assessed, and appropriate statistical methods were applied to address any deviations. Outliers were identified using Grubbs method with a significance level of *p* < 0.05. (Outliers: Figure 1A,B—1 mouse; Figure 4B,C—1 mouse; Figure 5A,E,F—1 mouse; Figure 5J—2 mice).

## 3. Results

### 3.1. Mouse Brain Gtf2i and Tfii-i Expression Levels Are Significantly Reduced in Early Post-Natal Stages

To define the post-natal developmental stage at which manipulation of *Gtf2i* expression has the greatest potential to be effective, we first characterized *Gtf2i* transcript levels, as well as those of the Tfii-i protein product, in the brains of naïve mice at different ages during the first post-natal month. We observed a progressive decline in *Gtf2i* mRNA levels from post-natal day 1 (P1) to P7, followed by a significant reduction starting at P14 relative to mRNA levels at P1 (Figure 1A). Tfii-i levels presented a similar temporal pattern (Figure 1B). These findings indicated that down-regulating *Gtf2i* expression within the first 14 post-natal days would be most effective, prompting us to systematically inject a virus that would affect *Gtf2i* levels in the early post-natal stage.

### 3.2. Systemic Viral Administration Results in a High Brain-Wide Infection Rate

In the present study, assessing the infection efficiency of different AAVs demonstrated that the AAV-PHP.eB serotype exhibited a superior central nervous system (CNS) infection ratio when compared to AAV9 [30] or PHP.B [31]. To ensure enhanced specificity and to target predominantly excitatory neurons in the forebrain, we employed the αCaMKII promoter, which displayed relatively high specificity relative to other promoters [31]. The αCaMKII promoter is primarily expressed in the mouse forebrain [32], first appearing in the middle of post-natal week three [33]. Therefore, employing an AAV-PHP.eB virus containing the αCaMKII promoter and a Cre-recombinase sequence allowed for targeted deletion of the *Gtf2i* gene from excitatory neurons in our mouse model.

To manipulate post-natal neuronal *Gtf2i* levels, an AAV-PHP.eB virus expressing a fluorophore and Cre-recombinase sequence under the regulation of the αCaMKII promoter was injected into the facial vein of *Gtf2i*(f/f) mice at P1–P2 (Figure 2A). The control group was injected with a similar virus not expressing the Cre-recombinase (Figure 2A). To evaluate transduction efficiency 21 days post-injection, we performed immunostaining of mouse brain sections using antibodies against NeuN to label all neurons and antibodies against mCherry to enhance the fluorescent signal of the infected cells (Figure 2B). The number of cells expressing both NeuN and mCherry was quantified and normalized to the total number of NeuN-expressing cells so as to calculate the percentage of infected neurons in the neuronal population. This quantification enabled us to determine the optimal titer and volume of virus needed to realize the desired infection properties.

We found that by injecting 30 µL of 10^12^ vg/mL of virus, we were able to achieve the highest infection ratio, with about 40% of the neuronal population being infected (Figure 2C). While perfusing the injected mice, we noticed purple coloring of the side of the brain where facial vein injection took place, suggesting accumulation of the virus. To address the uneven distribution of the virus, we injected half of the total volume originally injected to each side of the injected pup’s face. Upon assessing the number of cells labeled by both the anti-NeuN and anti-mCherry antibodies in the Cre-positive and the control groups, no significant differences were seen (Figure 2D), indicating comparable infection efficiencies. The distribution of viral infection throughout the brain, as depicted in Figure 2E, closely resembled the expression pattern of the αCaMKII promoter, as previously reported [32], as well as the expression pattern of the NEX promoter used in our previous study using the pre-natal *Gtf2i* deletion model [9].

**Figure 3:**
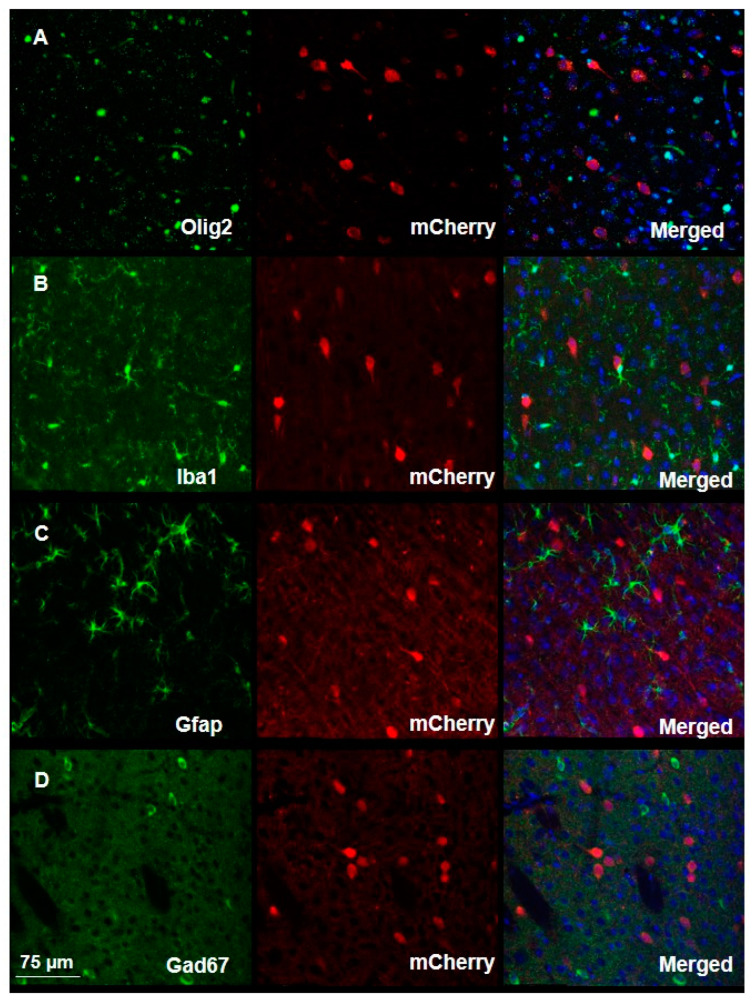
Viral transduction assessment demonstrates high specificity in the mouse brain. Immunofluorescent staining of brain slices from infected mice shows the high specificity of viral transduction, as demonstrated by the absence of co-localization of the mCherry signal and of signals associated with the binding of antibodies against (**A**) Olig2, (**B**) Iba1, (**C**) Gfap, or (**D**) Gad67. Merged images show cellular marker, viral mCherry and DAPI.

To assess the specificity of the virus, we conducted additional immunostaining using antibodies against specific cell markers, including anti-Iba1 antibodies to identify microglia, anti-Olig2 antibodies to identify oligodendrocytes, anti-Gfap antibodies to identify astrocytes, and anti-Gad67 antibodies to identify inhibitory neurons. Such analysis revealed the virus exhibits high specificity without transduction into any of the above-mentioned cell types (Figure 3, Supplementary Figure S1).

### 3.3. Systemic Post-Natal Viral Injection Effectively Reduces Gtf2i mRNA and Tfii-i Protein Levels

**Figure 4:**
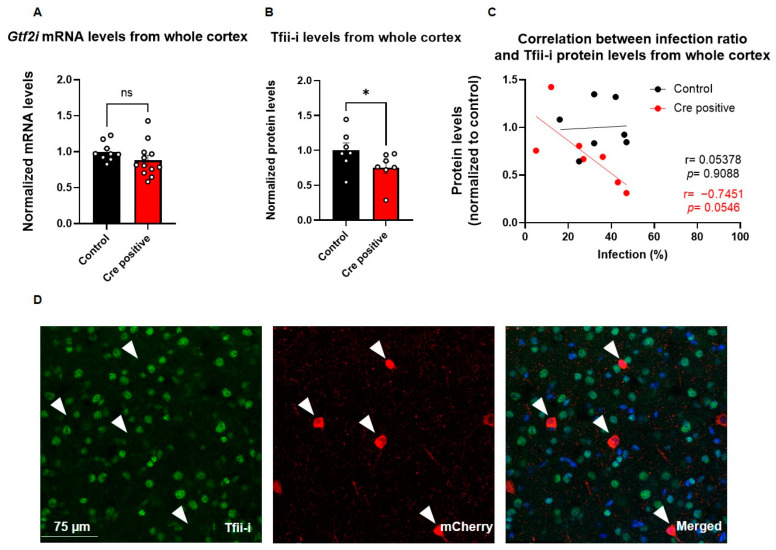
Viral injection led to a successful deletion of *Gtf2i* and reduced Tfii-i expression, specifically in excitatory neurons. (**A**) *Gtf2i* mRNA expression levels in the whole cortex of mice injected with the Cre-positive virus were lower than controls, although not significantly so. (**B**) Tfii-i protein levels in the whole cortex of Cre-positive injected mice were significantly lower than controls. (**C**) High correlation between the infection ratio and Tfii-i levels in the Cre-positive group, indicative of decreased Tfii-i expression as the infection rate increased but not in the control group. (**D**) Immunofluorescent staining of brain slices shows that cells infected with virus (red) do not express Tfii-i (green). Merged image shows Tfii-i, viral mCherry, and DAPI. Arrowheads mark cells positive for mCherry. Statistical significance was measured using (**A**) unpaired *t*-test: *n* = 9 controls, 12 Cre-positive, *p* = 0.2035; (**B**) Mann–Whitney test: *n* = 7 controls, 7 Cre-positive, *p* = 0.0379; and (**C**) correlation test: *n* = 7 controls, 7 Cre-positive, r = 0.05378, *p =* 0.9088 for controls, and r = −0.7451, *p* = 0.0546 for Cre-positive. * *p* < 0.05.

To evaluate the impact of Cre-recombinase-mediated excision of *Gtf2i*, we quantified *Gtf2i* mRNA and Tfii-i protein levels in the whole cortex of P60 mice. While *Gtf2i* mRNA levels showed only a trend of reduced expression in mice injected with the Cre-positive virus, as compared to controls (Figure 4A), the level of Tfii-i protein was significantly reduced (Figure 4B). The interplay between viral transduction and effective protein level reduction was demonstrated by the high correlation between Tfii-i levels and the infection ratio (Figure 4C), although not significantly. In contrast, such correlation was not observed in the control group, further highlighting the specific impact of the Cre-positive virus on Tfii-i expression.

To evaluate the successful deletion of *Gtf2i* in infected cells at single-cell resolution, we performed immunostaining for mCherry (indicating virus infection) and Tfii-i (Figure 4D). Notably, there was no co-localization of Tfii-i and mCherry, indicating that cells infected with the Cre-positive virus did not express Tfii-i (Figure 4D), further supporting the effectiveness of the deletion. All cells that were infected with the Cre-positive virus showed no expression of Tfii-i, indicating efficient knockout of *Gtf2i* following viral infection (Supplementary Figure S2).

### 3.4. Post-Natal Deletion of Gtf2i from Excitatory Neurons Results in Increased Sociability and Altered Anxiety-like Behavior

**Figure 5:**
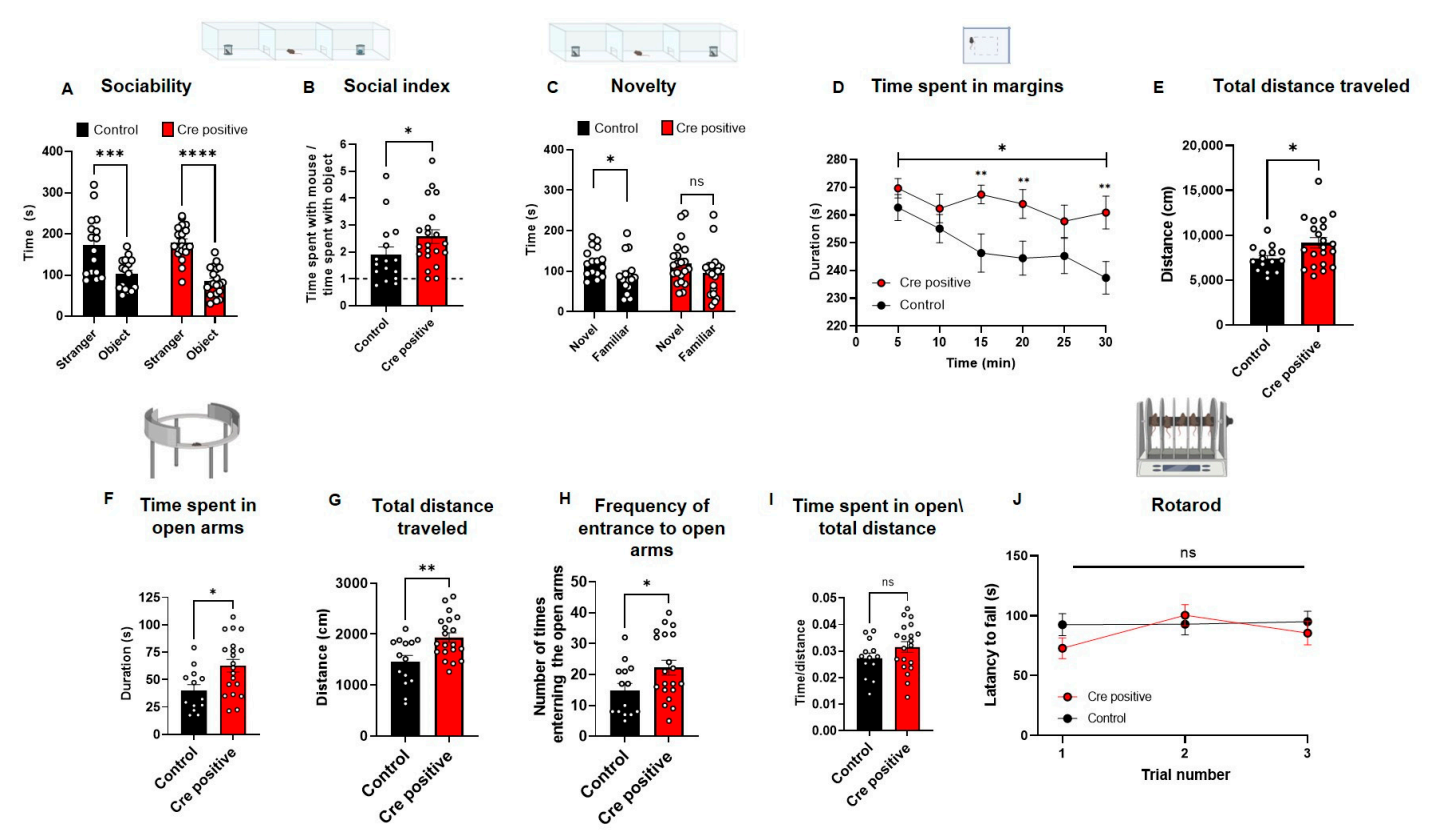
Post-natal neuronal *Gtf2i* deletion leads to hyper-sociability, altered anxiety-like behavior, and hyper-mobility. Post-natal *Gtf2i* deletion leads to hyper-sociability, defective cognition, increased anxiety, and ADHD-like behaviors but does not affect motor skills. (**A**) Cre-positive and control groups preferred spending more time with the stranger mouse. (**B**) The social index score of the Cre-positive group was significantly higher than that of the control group. (**C**) While the control group showed preference towards the novel stimulus, the Cre-positive did not seemingly differentiate between novel and familiar stimuli. (**D**) The Cre-positive group spent significantly more time on the margins of the open field arena, pointing to higher anxiety. (**E**) The Cre-positive group traveled significantly greater distances in the OF test, suggesting ADHD-like behavior. (**F**) In the EZM, the Cre-positive group spent significantly more time in the open arms. (**G**) The Cre-positive group traveled significantly longer distances in the EZM test. (**H**) The Cre-positive group entered and exited the open arms of the EZM significantly more times than did control mice. (**I**) Dividing the time spent in open arms by the total distance traveled revealed no significant difference between both groups, suggesting that the hyper-activity of the Cre-positive might mask any anxiety effect. (**J**) No differences in the motor skills of the groups were observed, as measured by the rotarod test. *n* = 16 control, 21 Cre-positive mice. Statistical significance was measured using (**A**) Two-way ANOVA with multiple comparisons; *p* = <0.0001 for both groups, F (1, 68) = 51.70. Šídák’s multiple comparisons test was used for post hoc analysis. (**B**) Unpaired t-test; *p* = 0.0374. (**C**) Paired *t*-test; *p =* 0.0400 (control). Wilcoxon matched-pairs signed-rank test; *p* = 0.1373 for Cre-positive mice. (**D**) Two-way ANOVA; treatment *p* = 0.0105, F (1, 35) = 7.304. Unpaired t-test for each 5 min time interval *p =* 0.2356, 0.1185, 0.0029, 0.1593, 0.0090 for 5, 10, 20, 25, 30 min intervals, respectively, and Mann Whitney test for 15 min time interval *p =* 0.0056. (**E**–**I**) Unpaired t-test; *p* = 0.0247, 0.0112, 0.0041, 0.0377, 0.1640. (**J**) Two-way ANOVA; treatment *p* = 0.4558, F (1, 32) = 0.5700; Šídák’s multiple comparisons test was used for post hoc analysis. * *p* < 0.05, ** *p* < 0.01, *** *p* < 0.005, **** *p* < 0.0001.

To study the behavioral impact of post-natal *Gtf2i* deletion in excitatory neurons, a series of behavioral tests were conducted on mice starting at P30. To study social behavior, the three-chamber social interaction test was used [34,35]. In this test, tested mice are placed in an apparatus containing three chambers connected with doors. In one side of the arena, a stimulus mouse is placed in a small wired cage, while on the opposite side of the arena, an inanimate stimulus is placed in a wired cage. For 15 min, the tested mouse is free to travel between the chambers and the time spent with each stimulus is measured. Mice injected with the Cre-positive virus exhibited increased sociability (Figure 5A), a prominent phenotype observed in individuals with WS [3] and previously associated with decreased Tfii-i expression levels [3,17].

Both tested groups demonstrated sociability by spending significantly more time with the stimulus mouse than with the object (Figure 5A), as expected, considering the general social tendencies of rodents [36,37]. Nevertheless, the social index, calculated as the time the test mouse spent in close interaction with the stimulus mouse divided by the time the test mouse spent in close interaction with the inanimate object, indicated that the Cre-positive mice preferred the social stimulus over the object significantly more than the control group’s preference (Figure 5B).

In the second part of the three-chambers test, mice are tested for their social novelty recognition. This is performed by replacing the inanimate object with a new stimulus mouse, and the test mouse is free to move around the arena for 15 min, in which the time the tested mouse spends with each of the mice is measured. Testing mice for their social novelty recognition, which can provide insight into the cognitive abilities of a mouse, revealed that the control group exhibited a significant preference for the novel mouse over the familiar one, whereas the Cre-positive group did not display such preference (Figure 5C).

To study whether anxiety-like behavior was also affected by the gene manipulation, especially given that increased non-social-related anxiety is a prominent behavioral phenotype in WS [38,39,40], we performed an open-field exploration test (OF). In this test, the tested mouse is placed in a 40 × 40 cm box and its exploration is tracked for 30 min. Mice injected with the Cre-positive virus spent significantly more time on the margins of the arena, as compared to controls, with behavior reflecting a heightened level of anxiety (Figure 5D). To measure mobility, we quantified the total distance traveled in the test and found that the Cre-positive group traveled significantly longer distances in the arena as compared to controls (Figure 5E). However, in the elevated zero maze (EZM), an additional test for anxiety-like behavior, the Cre-positive group spent significantly more time in the open arms of the maze than did the controls (Figure 5F). This test further showed the Cre-positive group presented higher mobility levels, indicated by the significantly longer total distances traveled (Figure 5G) and the significantly higher number of entries into the open arms of the maze (Figure 5H), a phenotype previously observed in attention-deficit/hyperactivity disorder (ADHD) mouse models [41]. Nevertheless, the increased time spent in the open arms of the maze by the Cre-positive group was not due to the increased mobility of this group (Figure 5I). The increased mobility we observed prompted us to measure whether this was the result of differences in motor coordination and gross motor skills, motor deficits also observed in WS [42,43]. Accordingly, we tested mice in the rotarod test and found no significant differences in motor coordination between the Cre-positive and control groups (Figure 5J).

### 3.5. Post-Natal Neuronal Gtf2i Deletion Does Not Affect Gross Anatomical Properties of the Brain or Cellular and Transcriptional Properties

**Figure 6:**
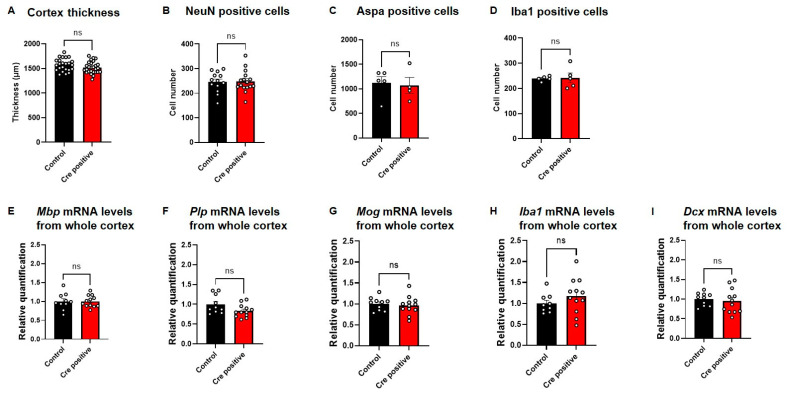
*Gtf2i* deletion does not lead to structural, cellular, or molecular differences between Cre-positive and control mice. (**A**) Cortex thickness in P60 mice in micrometers, as measured using ImageJ (V1.53K). (**B**) Number of neurons, measured as cells positive for a NeuN marker. (**C**) Number of myelinating oligodendrocytes, measured as cells positive for an ASPA marker. (**D**) Number of microglia, measured as cells positive for an Iba1 marker. Normalized (**E**) *Mbp*, (**F**) *Plp* (**G**) *Mog*, (**H**) *Iba1* and (**I**) *Dcx* mRNA levels in whole cortex of P45–60 mice injected with Cre-positive or control virus. Statistical significance was measured using (**A**,**B**,**D**,**E**–**I**) an unpaired *t*-test and (**C**) a Mann–Whitney test. (**A**) *n* = 24, 27, *p* = 0.0942. (**B**) *n* = 13, 16, *p* = 0.8977. (**C**) *n* = 5, 4, *p* = 0.7302. (**D**) *n* = 5, 5, *p* = 0.8770. (**E**) *n* = 10, 12, *p* = 0.9817. (**F**) *n* = 10, 12, *p* = 0.0621. (**G**) *n* = 10, 12, *p* = 0.6557. (**H**) *n* = 10, 12, *p* = 0.2714. (**I**) *n* = 10, 12, *p* = 0.6913 for control and Cre-positive, respectively.

To evaluate whether the neuronal *Gtf2i* deletion after the period of embryonic brain development affected cortical development, we measured the thickness of the cortex and found no significant difference between both groups (Figure 6A). Furthermore, no significant differences were found in the number of neurons (Figure 6B), myelinating oligodendrocytes (Figure 6C), and microglia cells (Figure 6D) in the mouse cortex.

We previously showed that pre-natal deletion of *Gtf2i* from excitatory neurons affects the expression level of myelination-related mRNA transcripts [9]. To assess whether post-natal neuronal *Gtf2i* deletion also affects transcripts related to myelination, we performed quantitative (q)PCR to quantify transcripts level in the mouse cortex. No significant differences in *Mbp* (Figure 6E), *Plp* (Figure 6F) and *Mog* (Figure 6G) mRNA levels were found between the two groups. Similarly, no significant differences were measured in the mRNA levels of the microglial transcript *Iba1* (Figure 6H) and the neuronal migration factor *Dcx* (Figure 6I).

## 4. Discussion

In this study, we elicited a post-natal deletion of *Gtf2i* from excitatory neurons using IV injections of AAV-PHP.eB expressing Cre-recombinase under the αCaMKII promoter. The deletion led to increased levels of social behavior, anxiety-like behavior, and hyper-mobility compared to controls.

Our rationale for examining such post-natal deletion stemmed from the high expression levels of the transcript and protein product in the first two post-natal weeks. Since pre-natal deletion of *Gtf2i* resulted in behavioral changes [9], we asked whether post-natal *Gtf2i* deletion would yield similar outcomes.

Many studies have demonstrated the diverse regulatory capabilities of transcription factors (TFs) in gene expression [44,45,46,47,48]. Specifically, these TFs have been shown to either enhance or inhibit the expression of genes, and in some cases, they exhibit both functions concurrently [48].

One prominent TF exhibiting such dual activity is Tfii-i [10,49]. For instance, during embryonic development, Tfii-i has been observed to enhance the expression of the *Vegfr2* gene [10]. On the other hand, Tfii-i variants exhibit distinct functions in signal-induced transcription regulation; specifically, the β isoform suppresses c-Fos transcription, whereas the Δ isoform acts as an enhancer in murine fibroblasts [50,51].

Revealing *Gtf2i*’s expression pattern during embryonic and early post-natal development is of great significance based on these findings. If Tfii-i has an enhancing effect on genes critical to embryonic development, it would necessitate higher expression levels during relevant stages. Conversely, if Tfii-i exerts an inhibitory effect on brain development-promoting transcripts, lower expression levels would be expected during periods of enhanced brain development.

To ensure that our research aligned with previous knowledge regarding the effects of pre-natal deletion of *Gtf2i* in excitatory neurons, we employed the αCaMKII promoter and observed a similar infection pattern to that reported in the pre-natal model using the NEX-Cre line [9], as well as the expression pattern of the αCaMKII promoter, as previously described [32]. The fact that a manipulation through exogenous transgenesis via IV administration results in transfection pattern similar to the basal expression of αCaMKII in the mouse brain further validates the reliability, efficacy, and specificity of this method. The similarity between the two models enabled us to compare the results of both studies and elucidate the predominantly pre-natal and post-natal effects of *Gtf2i*. Determining the critical time window for different phenotypes provides better understanding of the developmental roles of this gene and how gene therapy could impact key phenotypes.

Our study found that social behavior was affected by both post-natal and pre-natal *Gtf2i* deletion from excitatory neurons, which resulted in hyper-sociability. However, in the pre-natal model, motor deficits were observed in the conditional knock-out (cKO) group [9], whereas in the present study, motor impairments were not observed following gene deletion. Similar findings were obtained in other studies conducted in our laboratory that aimed to ameliorate symptoms of WS and autism spectrum disorder (ASD) in appropriate mouse models using post-natal approaches, such as hyperbaric oxygen therapy (HBOT) [52] and drug administration. These social findings suggest that the critical time window for influencing social behavior is not specific, whereas acting during the pre-natal period appears to be more crucial for affecting motor skills.

In the second part of the three-chambered test, as described before, the Cre-positive mice did not exhibit differences in their responses to novel or familiar stimuli. Furthermore, in both the OF and EZM tests, the mice displayed higher mobility and greater total distances traveled. These findings suggest that while the Cre-positive mice demonstrated high sociability levels, they lacked the ability to differentiate between familiar and novel stimuli, indicating possible cognitive impairment. This phenotype is consistent with WS [1,5,53]. The fact that pre-natal *Gtf2i* deletion from excitatory neurons did not result in increased mobility in the current study suggests that *Gtf2i* plays a post-natal hyper-activity-mediating role.

Insights from both human and animal model studies suggest that an imbalance in excitatory-inhibitory (E/I) neuronal activity could potentially underlie the physiological mechanism behind these abnormalities [54]. Disruptions in the E/I balance have been associated with either hyperactivity [55] or hypoactivity [56] in specific brain regions essential for behavior modulation. For instance, in mice, heightened excitatory activity in the medial prefrontal cortex has been linked to impaired social behavior [57]. Interestingly, supporting the E/I imbalance theory, increased activation of inhibitory cells has been found to rescue the social deficits in these mice [57]. Also, previous studies suggested that one of the causes of ADHD might be E/I imbalance in the fronto-striatal circuitry [58]. This might indicate that by deleting *Gtf2i* from excitatory neurons we affected the E/I neural activity which in turn supported the abnormal social behavior and ADHD phenotypes. To further study this, future studies should characterize the consequences of *Gtf2i* deletion from inhibitory neurons also expected to affect the E/I balance.

Our study further indicated that post-natal deletion of *Gtf2i* from excitatory neurons did not affect cortical development, cellular properties, or transcription regulation. This is in contrast to the deficits in these parameters we previously described upon pre-natal deletion [9]. A limitation of our study was the incomplete infection of the entire neuronal population by the virus, which hindered the comprehensive interpretation of gene deletion effects across all cells. Our manipulation resulted in an average infection ratio of 40% of the neuronal population in the cortex. Although this level of infection post-natally may be adequate to induce behavioral changes, it might not be sufficient to detect significant alterations at the cellular or transcriptomic levels.

Nonetheless, our findings support a key role for *Gtf2i* in embryonic brain development [10]. Our results also suggest that *Gtf2i* plays a crucial role in the functioning of the brain and nervous system, especially those regions mediating behavior. The altered behavior seen upon *Gtf2i* deletion in the current study indicates that the affected behaviors are controlled by neural circuits that contain excitatory neurons in which *Gtf2i* is essential, even if *Gtf2i* levels were normal during embryonic development. Still, our understanding of the post-natal roles of *Gtf2i* is still evolving, with further research being needed to uncover the full extent of its function during this period.

Current treatments of neurodevelopmental disorders involve a combination of behavioral therapies, cognitive interventions, and pharmaceutical agents to target the predominant symptoms [59]. Our study revealed that post-natal neuronal deletion of *Gtf2i* elicits several phenotypes also observed upon pre-natal *Gtf2i* deletion, including hyper-sociability, indicating the potential applicability of post-natal gene manipulation to ameliorate behavioral deficits known to be affected in neurodevelopmental disorders, such as ASD. Because *Gtf2i* role is dosage-dependent [1,9,12,13,14,15,16,17,18], manipulating its expression levels in different cell populations, or in different dosages, might be of interest for future research. Furthermore, our findings advance our understanding of post-natal *Gtf2i* roles, specifically in neurons, and the regulation of *Gtf2i* expression in the mouse brain during post-natal development.

## Figures and Tables

**Table 1:** Primers used in this study.

Name of Primer	Sequence
Nex-Cre fwd	GAGTCCTGGAATCAGTCTTTTTC
Nex-Cre rev	AGAATGTGGAGTAGGGTGAC
Nex-Cre KO	CCGCATAACCAGTGAAACAG
Rmb31x/y fwd	CACCTTAAGAACAAGCCAATACA
Rmb31x/y rev	GGCTTGTCCTGAAAACATTTGG
*Gapdh* fwd	GCCTTCCGTGTTCCTACC
*Gapdh* rev	CCTCAGTGTAGCCCAAGATG
*Gtf2i* fwd	GGTCACCTCAACTTCGGGG
*Gtf2i* rev	AGGCCCTTCTGAAACTGATGG
*Iba1* fwd	TCTGCCGTCCAAACTTGAAG
*Iba1* rev	GTTTCTCCAGCATTCGCTTC
*Plp* fwd	TTGTTTGGGAAAATGGCTAGGAC
*Plp* rev	TGGTCCAGGTATTGAAGTAAATG
*Mbp* fwd	CCAGAGTGAAAACCCAGTAGTCC
*Mbp* rev	TGTCTCTTCCTCCCCAGCTAAA
*Mog* fwd	AGCTGCTTCCTCTCCCTTCTC
*Mog* rev	ACTAAAGCCCGGATGGGATAC
*Dcx* fwd	CATCACAGAAGCGATCAAACTGGA
*Dcx* rev	CAGGACCACAAGCAATGAACACA

## Data Availability

Data are contained within the article or Supplementary Material.

## Supplementary Materials

The following supporting information can be downloaded at: https://www.mdpi.com/article/10.3390/biomedicines11082273/s1, Figure S1: Quantitative analysis of viral infection specificity. Cells positive for any of the following markers were quantified (red circles), as well as cells positive both for the marker and for mCherry (Gad67 for inhibitory neurons, Gfap for astrocytes, Iba1 for microglia and Olig2 for oligodendrocytes); Figure S2: Quantitative analysis of viral infection efficacy. In all the Cre positive infected cells, no Tfii-i expression was measured.

## References

[1] Kozel B.A., Barak B., Kim C.A., Mervis C.B., Osborne L.R., Porter M., Pober B.R. (2021). Williams syndrome. Nat. Rev. Dis. Primers.

[2] Barak B., Feng G. (2016). Neurobiology of social behavior abnormalities in autism and Williams syndrome. Nat. Neurosci..

[3] Pober B.R. (2010). Williams-Beuren syndrome. N. Engl. J. Med..

[4] Dai L., Bellugi U., Chen X.N., Pulst-Korenberg A.M., Jarvinen-Pasley A., Tirosh-Wagner T., Eis P.S., Graham J., Mills D., Searcy Y. (2009). Is it Williams syndrome? GTF2IRD1 implicated in visual-spatial construction and GTF2I in sociability revealed by high resolution arrays. Am. J. Med. Genet. Part A.

[5] Morris C.A., Mervis C.B., Hobart H.H., Gregg R.G., Bertrand J., Ensing G.J., Sommer A., Moore C.A., Hopkin R.J., Spallone P.A. (2003). *GTF2I* hemizygosity implicated in mental retardation in Williams syndrome: Genotype-phenotype analysis of five families with deletions in the Williams syndrome region. Am. J. Med. Genet. Part A.

[6] Martin L.A., Iceberg E., Allaf G. (2018). Consistent hypersocial behavior in mice carrying a deletion of *Gtf2i* but no evidence of hyposocial behavior with *Gtf2i* duplication: Implications for Williams-Beuren syndrome and autism spectrum disorder. Brain Behav..

[7] Sakurai T., Dorr N.P., Takahashi N., McInnes L.A., Elder G.A., Buxbaum J.D. (2011). Haploinsufficiency of *Gtf2i*, a gene deleted in Williams Syndrome, leads to increases in social interactions. Autism Res..

[8] Osborne L.R. (2010). Animal models of Williams syndrome. Am. J. Med. Genet. Part C Semin. Med. Genet..

[9] Barak B., Zhang Z., Liu Y., Nir A., Trangle S.S., Ennis M., Levandowski K.M., Wang D., Quast K., Boulting G.L. (2019). Neuronal deletion of *Gtf2i*, associated with Williams syndrome, causes behavioral and myelin alterations rescuable by a remyelinating drug. Nat. Neurosci..

[10] Enkhmandakh B., Makeyev A.V., Erdenechimeg L., Ruddle F.H., Chimge N.O., Tussie-Luna M.I., Roy A.L., Bayarsaihan D. (2009). Essential functions of the Williams-Beuren syndrome-associated TFII-I genes in embryonic development. Proc. Natl. Acad. Sci. USA.

[11] Farran E.K., Karmiloff-Smith A. (2012). Neurodevelopmental Disorders across the Lifespan: A Neuroconstructivist Approach.

[12] Ewart A.K., Morris C.A., Atkinson D., Jin W.S., Sternes K., Spallone P., Stock A.D., Leppert M., Keating M.T. (1993). Hemizygosity at the Elastin Locus in a Developmental Disorder, Williams-Syndrome. Nat. Genet..

[13] Morris C.A., Mervis C.B., Paciorkowski A.P., Abdul-Rahman O., Dugan S.L., Rope A.F., Bader P., Hendon L.G., Velleman S.L., Klein-Tasman B.P. (2015). 7q11.23 Duplication syndrome: Physical characteristics and natural history. Am. J. Med. Genet. Part A.

[14] Mervis C.B., Klein-Tasman B.P., Huffman M.J., Velleman S.L., Pitts C.H., Henderson D.R., Woodruff-Borden J., Morris C.A., Osborne L.R. (2015). Children with 7q11.23 duplication syndrome: Psychological characteristics. Am. J. Med. Genet. Part A.

[15] Mervis C.B., Dida J., Lam E., Crawford-Zelli N.A., Young E.J., Henderson D.R., Onay T., Morris C.A., Woodruff-Borden J., Yeomans J. (2012). Duplication of *GTF2I* results in separation anxiety in mice and humans. Am. J. Hum. Genet..

[16] Osborne L.R., Mervis C.B. (2021). 7q11.23 deletion and duplication. Curr. Opin. Genet. Dev..

[17] Somerville M.J., Mervis C.B., Young E.J., Seo E.J., del Campo M., Bamforth S., Peregrine E., Loo W., Lilley M., Perez-Jurado L.A. (2005). Severe expressive-language delay related to duplication of the Williams-Beuren locus. N. Engl. J. Med..

[18] Pinelli M., Terrone G., Troglio F., Squeo G.M., Cappuccio G., Imperati F., Pignataro P., Genesio R., Nitch L., Del Giudice E. (2020). A small 7q11.23 microduplication involving *GTF2I* in a family with intellectual disability. Clin. Genet..

[19] Mendell J.R., Al-Zaidy S.A., Rodino-Klapac L.R., Goodspeed K., Gray S.J., Kay C.N., Boye S.L., Boye S.E., George L.A., Salabarria S. (2021). Current Clinical Applications of In Vivo Gene Therapy with AAVs. Mol. Ther..

[20] Pupo A., Fernandez A., Low S.H., Francois A., Suarez-Amaran L., Samulski R.J. (2022). AAV vectors: The Rubik’s cube of human gene therapy. Mol. Ther. J. Am. Soc. Gene Ther..

[21] Daya S., Berns K.I. (2008). Gene therapy using adeno-associated virus vectors. Clin. Microbiol. Rev..

[22] Borralleras C., Sahun I., Perez-Jurado L.A., Campuzano V. (2015). Intracisternal *Gtf2i* Gene Therapy Ameliorates Deficits in Cognition and Synaptic Plasticity of a Mouse Model of Williams-Beuren Syndrome. Mol. Ther..

[23] Enkhmandakh B., Stoddard C., Mack K., He W., Kaback D., Yee S.P., Bayarsaihan D. (2016). Generation of a mouse model for a conditional inactivation of *Gtf2i* allele. Genesis.

[24] Goebbels S., Bormuth I., Bode U., Hermanson O., Schwab M.H., Nave K.A. (2006). Genetic targeting of principal neurons in neocortex and hippocampus of NEX-Cre mice. Genesis.

[25] Gombash Lampe S.E., Kaspar B.K., Foust K.D. (2014). Intravenous injections in neonatal mice. J. Vis. Exp. JoVE.

[26] Grad M., Nir A., Levy G., Trangle S.S., Shapira G., Shomron N., Assaf Y., Barak B. (2022). Altered White Matter and microRNA Expression in a Murine Model Related to Williams Syndrome Suggests That miR-34b/c Affects Brain Development via Ptpru and Dcx Modulation. Cells.

[27] Nir A., Barak B. (2021). White matter alterations in Williams syndrome related to behavioral and motor impairments. Glia.

[28] Tunster S.J. (2017). Genetic sex determination of mice by simplex PCR. Biol. Sex Differ..

[29] Schmittgen T.D., Livak K.J. (2008). Analyzing real-time PCR data by the comparative C(T) method. Nat. Protoc..

[30] Chatterjee D., Marmion D.J., McBride J.L., Manfredsson F.P., Butler D., Messer A., Kordower J.H. (2022). Enhanced CNS transduction from AAV.PHP.eB infusion into the cisterna magna of older adult rats compared to AAV9. Gene Ther..

[31] Radhiyanti P.T., Konno A., Matsuzaki Y., Hirai H. (2021). Comparative study of neuron-specific promoters in mouse brain transduced by intravenously administered AAV-PHP.eB. Neurosci. Lett..

[32] Burgin K.E., Waxham M.N., Rickling S., Westgate S.A., Mobley W.C., Kelly P.T. (1990). In situ hybridization histochemistry of Ca^2+^/calmodulin-dependent protein kinase in developing rat brain. J. Neurosci..

[33] Tsien J.Z., Chen D.F., Gerber D., Tom C., Mercer E.H., Anderson D.J., Mayford M., Kandel E.R., Tonegawa S. (1996). Subregion- and cell type-restricted gene knockout in mouse brain. Cell.

[34] Moy S.S., Nadler J.J., Perez A., Barbaro R.P., Johns J.M., Magnuson T.R., Piven J., Crawley J.N. (2004). Sociability and preference for social novelty in five inbred strains: An approach to assess autistic-like behavior in mice. Genes Brain Behav..

[35] Nadler J.J., Moy S.S., Dold G., Trang D., Simmons N., Perez A., Young N.B., Barbaro R.P., Piven J., Magnuson T.R. (2004). Automated apparatus for quantitation of social approach behaviors in mice. Genes Brain Behav..

[36] Vanderschuren L.J., Niesink R.J., Van Ree J.M. (1997). The neurobiology of social play behavior in rats. Neurosci. Biobehav. Rev..

[37] Vanderschuren L.J., Achterberg E.J., Trezza V. (2016). The neurobiology of social play and its rewarding value in rats. Neurosci. Biobehav. Rev..

[38] Dykens E.M. (2003). Anxiety, fears, and phobias in persons with Williams syndrome. Dev. Neuropsychol..

[39] Green T., Avda S., Dotan I., Zarchi O., Basel-Vanagaite L., Zalsman G., Weizman A., Gothelf D. (2012). Phenotypic psychiatric characterization of children with Williams syndrome and response of those with ADHD to methylphenidate treatment. American journal of medical genetics. Part B Neuropsychiatr. Genet..

[40] Zarchi O., Diamond A., Weinberger R., Abbott D., Carmel M., Frisch A., Michaelovsky E., Gruber R., Green T., Weizman A. (2014). A comparative study of the neuropsychiatric and neurocognitive phenotype in two microdeletion syndromes: Velocardiofacial (22q11.2 deletion) and Williams (7q11.23 deletion) syndromes. Eur. Psychiatry.

[41] Russell V.A. (2011). Overview of animal models of attention deficit hyperactivity disorder (ADHD). Curr. Protoc. Neurosci..

[42] Preus M. (1984). The Williams syndrome: Objective definition and diagnosis. Clin. Genet..

[43] Greer M.K., Brown F.R., Pai G.S., Choudry S.H., Klein A.J. (1997). Cognitive, adaptive, and behavioral characteristics of Williams syndrome. Am. J. Med. Genet..

[44] Stolt C.C., Rehberg S., Ader M., Lommes P., Riethmacher D., Schachner M., Bartsch U., Wegner M. (2002). Terminal differentiation of myelin-forming oligodendrocytes depends on the transcription factor Sox10. Genes Dev..

[45] Lee K.E., Nam S., Cho E.A., Seong I., Limb J.K., Lee S., Kim J. (2008). Identification of direct regulatory targets of the transcription factor Sox10 based on function and conservation. BMC Genom..

[46] Wang S., Sdrulla A., Johnson J.E., Yokota Y., Barres B.A. (2001). A role for the helix-loop-helix protein Id2 in the control of oligodendrocyte development. Neuron.

[47] Samanta J., Kessler J.A. (2004). Interactions between ID and OLIG proteins mediate the inhibitory effects of BMP4 on oligodendroglial differentiation. Development.

[48] Makeyev A.V., Bayarsaihan D. (2009). Alternative splicing and promoter use in TFII-I genes. Gene.

[49] Roy A.L. (2012). Biochemistry and biology of the inducible multifunctional transcription factor TFII-I: 10 years later. Gene.

[50] Shirai Y., Li W., Suzuki T. (2017). Role of Splice Variants of *Gtf2i*, a Transcription Factor Localizing at Postsynaptic Sites, and Its Relation to Neuropsychiatric Diseases. Int. J. Mol. Sci..

[51] Hakre S., Tussie-Luna M.I., Ashworth T., Novina C.D., Settleman J., Sharp P.A., Roy A.L. (2006). Opposing functions of TFII-I spliced isoforms in growth factor-induced gene expression. Mol. Cell.

[52] Fischer I., Shohat S., Levy G., Bar E., Trangle S.S., Efrati S., Barak B. (2022). Hyperbaric Oxygen Therapy Alleviates Social Behavior Dysfunction and Neuroinflammation in a Mouse Model for Autism Spectrum Disorders. Int. J. Mol. Sci..

[53] Crespi B.J., Hurd P.L. (2014). Cognitive-behavioral phenotypes of Williams syndrome are associated with genetic variation in the *GTF2I* gene, in a healthy population. BMC Neurosci..

[54] Baroncelli L., Braschi C., Spolidoro M., Begenisic T., Maffei L., Sale A. (2011). Brain plasticity and disease: A matter of inhibition. Neural Plast..

[55] Gogolla N., Takesian A.E., Feng G., Fagiolini M., Hensch T.K. (2014). Sensory integration in mouse insular cortex reflects GABA circuit maturation. Neuron.

[56] Peca J., Feliciano C., Ting J.T., Wang W., Wells M.F., Venkatraman T.N., Lascola C.D., Fu Z., Feng G. (2011). Shank3 mutant mice display autistic-like behaviours and striatal dysfunction. Nature.

[57] Yizhar O., Fenno L.E., Prigge M., Schneider F., Davidson T.J., O’Shea D.J., Sohal V.S., Goshen I., Finkelstein J., Paz J.T. (2011). Neocortical excitation/inhibition balance in information processing and social dysfunction. Nature.

[58] Mamiya P.C., Arnett A.B., Stein M.A. (2021). Precision Medicine Care in ADHD: The Case for Neural Excitation and Inhibition. Brain Sci..

[59] Levy G., Barak B. (2021). Postnatal therapeutic approaches in genetic neurodevelopmental disorders. Neural Regen. Res..

