# Supplementary material for: Deletion of Gtf2i via Systemic Administration of AAV-PHP.eB Virus Increases Social Behavior in a Mouse Model of a Neurodevelopmental Disorder
Source: Biomedicines. 2023 Aug 15;11(8):2273. doi: 10.3390/biomedicines11082273 (PMC10452363; doi:10.3390/biomedicines11082273)
Supplement: Supplementary file 1 [file biomedicines-11-02273-s001.zip › biomedicines-2519408-supplementary.pdf]

biomedicines

MDPI

**Supplementary figures:**

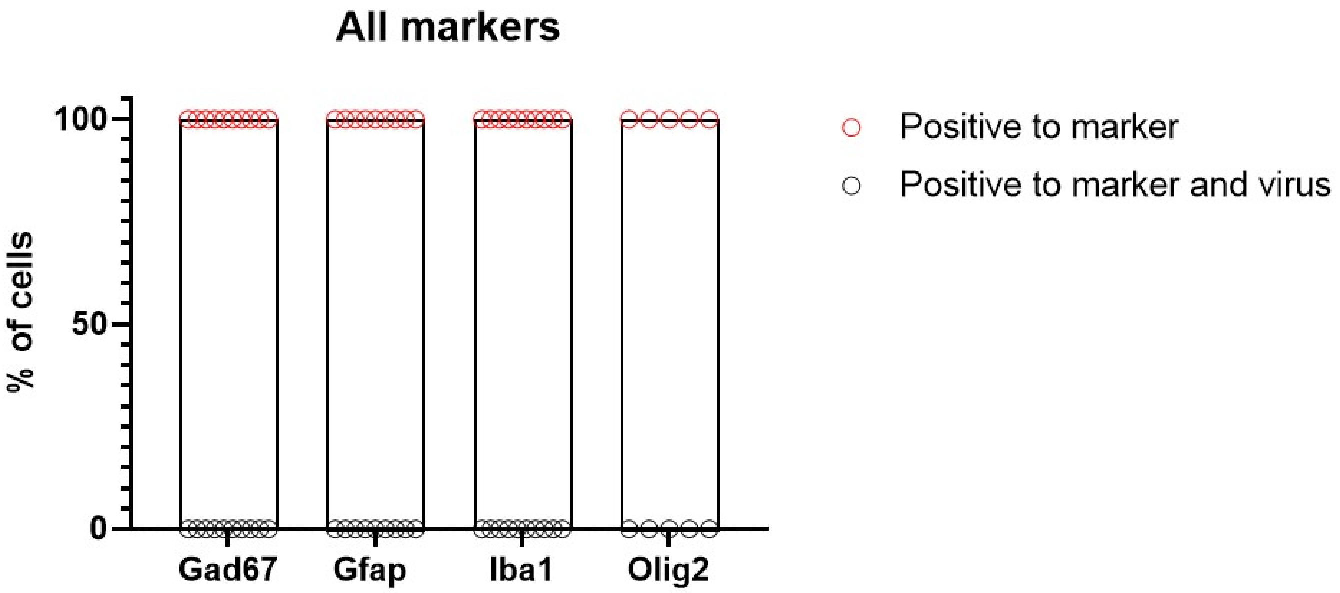


Supplementary Figure S1: Quantitative analysis of viral infection specificity. Cells positive for any of the following markers were quantified (red circles), as well as cells positive both for the marker and for mCehrry (Gad67 for inhibitory neurons, Gfap for astrocytes, Iba1 for microglia and Olig2 for oligodendrocytes).

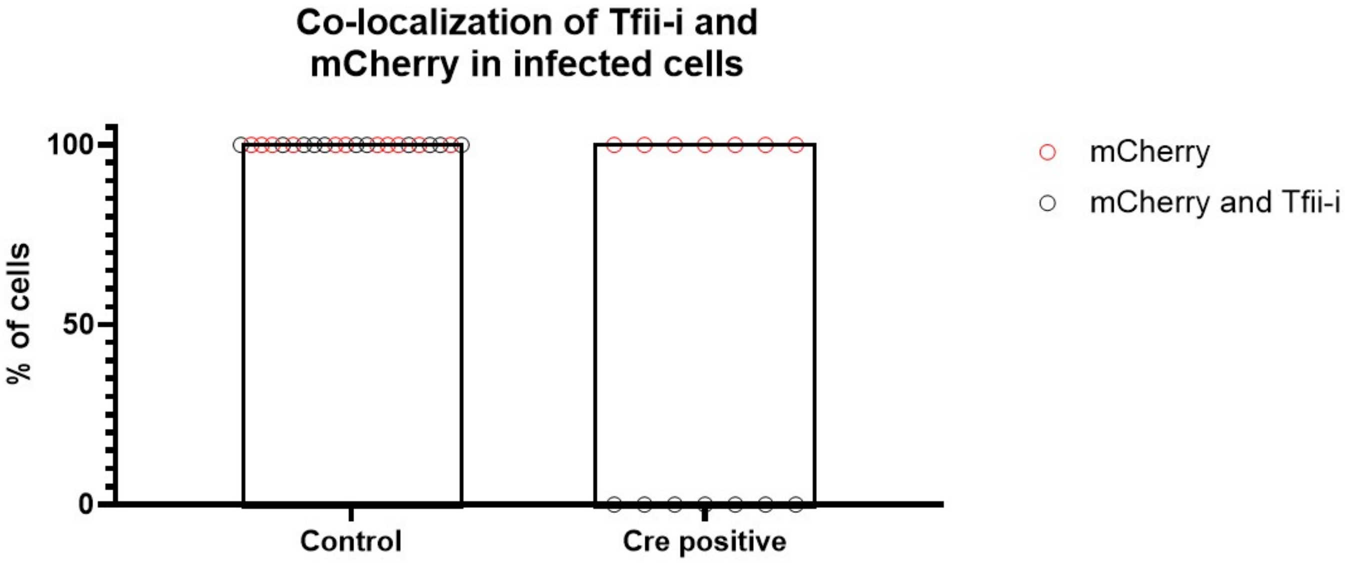


Supplementary Figure S2: Quantitative analysis of viral infection efficacy. In all the Cre positive infected cells, no Tfii-i expression was measured.
